# Have Mercy on Dumb Beasts1Read before the meeting of the Genesee Valley Veterinary Medical Association, January 12, 1899.

**Published:** 1899-07

**Authors:** J. B. Warner

**Affiliations:** President of the Rochester Humane Society, Rochester, N. Y.


### HAVE MERCY ON DUMB BEASTS.[1]

By J. B. Warner,

PRESIDENT OF THE ROCHESTER HUMANE SOCIETY, ROCHESTER, N. Y.

It was very kindly suggested to me, a few days ago, that an opportunity would be afforded me on this occasion to say

[1] Read before the meeting of the Genesee Valley Veterinary Medical Association, January 12, 1899.

a few words in behalf of the Humane Society of Rochester for the Prevention of Cruelty to Animals. I was a good deal surprised at the suggestion, as the few talents I possess are rather of a practical nature, and thus far have been devoted to the common everyday work of the society and not to the painting of word pictures which might, perhaps, induce others to join us in the good work. I doubted seriously whether I had the right to take up the valuable time of so intelligent a body of men with crude thoughts on a subject which deserves the attention of the best ability in the land. But the abuses and cruel treatment to which dumb animals in our midst are sometimes subjected move me greatly, and I am here to-day in the hope that by merely calling your attention to what our society is doing you will give us a larger measure of your friendship and support. In fact, your work as veterinarians is very closely allied to that of our society. You strive by the skilful use of medicine and surgery to relieve domestic animals from sickness and the various ills which afflict them, and thereby to restore them to usefulness and save their owners from loss. From a practical standpoint you save property from loss or depreciation, but incidentally you are doing the work of the humane society, which has no excuse for existence except its declared purpose to strive in every legitimate way to protect dumb animals from pain and suffering. There ought, then, to be a strong bond of sympathy between your association and our society, and I believe there is, and that it will strengthen as time shows that we can be of use to each other. Our society has always had the active support, and, as far as I know, the approval of the veterinarians of Rochester, and the help so freely given by them has been invaluable in the prosecution of our work.

As the existence of our society depends on the generosity and good will of the community, it is particularly desirable that we should have the indorsement of the veterinarians, who by reason of their calling, are better acquainted with the ills to which our dumb animals are subjected by thoughtless and vicious people, and hence in a better position to judge of the necessity of systematic effort to secure the enforcement of our laws against "the man who has no mercy on his beast." May we not hope, then, that you, a body of representative men from six of the finest counties in the State, will give the cause of "Prevention of Cruelty to Animals" your indorsement?

In our record-books at our animal shelter, on Falls Street, will be found an entry of every case of cruelty or animal suffering which the society has been called upon to investigate or relieve during the past year. This enables me to give exact figures showing what was accomplished.

Horses to the number of 126 were found by our agent being driven on the pavements either without shoes, or, if in winter, without sharp calks. One hundred and sixty were found unfit for work on account of lameness or other ailment, and sent to their stables. Thirty-two were found standing on the street without protection from the cold, and their owners induced to provide blankets. Our agents examined 1032 horses and mules in use on the Erie Canal, and in all cases of neglect or abuse relief was obtained as far as practicable. Under the advice of Drs. Drinkwater and McKenzie, who have so generously given their services to the society, 72 horses were put to death to relieve them from their sufferings.

Seven hundred and three sick, worthless, or stray dogs and cats were collected from various parts of the city, and accorded easy deaths in our gas chamber.

In addition to all this, 679 cases of actual cruelty were reported to the society, and all properly investigated, involving a large amount of work for our attorney and other officers.

In all 2804 cases were looked after by the society during the year.

This brief statement will give you some idea of our work and enable you to form an estimate of the amount of suffering which has been relieved through our efforts.

As individuals you can help the cause greatly, in your respective localities, in various ways. And here, perhaps, it would be well to explain that the charter of the Rochester Society limits its jurisdiction to Monroe County, but that the American Society for Prevention of Cruelty to Animals, with headquarters in New York City, has jurisdiction throughout the State, and is prepared to act in any county where there is no local society. This New York Society is supposed to have a regularly appointed agent in each county of the State, but in many cases where there is no local interest taken in humane work he is never called upon to act. Unfortunately, cruelty to animals is not confined to any one locality, but is found in greater or less degree in every community, and the only effective remedy is found in the enforcement of the law through local agents. Experience has taught us that the existence of such an agent, especially if backed by the better element of the neighborhood, acts as a "preventive," and inspires respect for law in the evil-minded. Is it not a pity, then, that a few persons cannot be found in every town and hamlet of the State to give a little of their time and attention to a cause which seeks not only to punish evildoers, but to encourage the growth of that divine attribute which we call mercy, compassion for God's creatures in misfortune "whether brute or human."

As I said above, you can each of you accomplish much good in your own locality by interesting a few of your friends and neighbors in this subject, and so start the ball rolling which may result in the formation of a society—either independent or adjunct to the American Society of New York City. If cases of cruelty come to your notice which seem to demand the intervention of law, you can report them, if in this county to us, and if in some other county to the American Society in New York. In all such cases you will find that your communication will be attended to promptly and that your name will not be used in a way to cause you annoyance or embarrassment.

In the event of your wanting information in regard to the formation of a society, or the laws under which we act, it will be cheerfully given, by us, on application.

